# Mycobacterium camsae sp. nov. and Mycobacterium pumcae sp. nov., two species isolated from human skin infection

**DOI:** 10.1099/ijsem.0.006960

**Published:** 2025-11-14

**Authors:** Youming Mei, Wenyue Zhang, Ying Shi, Haiqin Jiang, Qian Zhang, Tian Gan, Jiayi Peng, Huan Mei, Suo Mo, Hongsheng Wang

**Affiliations:** 1 Hospital for Skin Diseases, Institute of Dermatology, Chinese Academy of Medical Sciences & Peking Union Medical College, Nanjing, PR China; 2 Jiangsu Provincial Key Laboratory of Dermatology, Nanjing, PR China; 3 Center for Global Health, School of Public Health, Nanjing Medical University, Nanjing, Jiangsu, PR China; 4 School of Population Medicine and Public Health, Chinese Academy of Medical Sciences and Peking Union Medical College, Beijing, PR China

**Keywords:** bacterial WGS, whole genome sequencing, *Mycobacterium*, *Mycobacterium gordonae *complex, nontuberculous mycobacteria

## Abstract

Two slow-growing nontuberculous mycobacterial strains were isolated from clinical skin lesions in Jiangsu, PR China, and identified as novel species within the genus *Mycobacterium*. The bacterial colonies exhibited scotochromogenic pigmentation and rod-shaped morphology, and they were classified as acid-fast bacilli. Phylogenetic analysis indicated that the two strains were closely related to *Mycobacterium gordonae*. The two strains, X7091^T^ and Z3061^T^, exhibited average nucleotide identity and *in silico* DNA–DNA hybridization values below the threshold for species delineation when compared with other type strains of *M. gordonae* complex. Therefore, two novel *Mycobacterium* species are proposed: *Mycobacterium camsae* sp. nov., with the type strain being X7091^T^ (=CGMCC 1.90336^T^=JCM 37414^T^), and *Mycobacterium pumcae* sp. nov., with the type strain being Z3061^T^ (=CGMCC 1.90337^T^=JCM 37415^T^).

## Introduction

Nontuberculous mycobacteria are ubiquitous saprophytic organisms widely distributed in natural and artificial environments, including water and soil. The majority are classified as opportunistic pathogens. Technological advances in whole-genome sequencing generated vast amounts of genomic data, facilitating genome-based taxonomic analyses and enabling the robust discovery of novel species. The taxon *Mycobacterium* Lehmann and Neumann 1896 (Approved Lists 1980) currently includes 203 species with valid published names, as recorded in the List of Prokaryotic names with Standing in Nomenclature (https://lpsn.dsmz.de/genus/mycobacterium; 23 May 2025) [1].

The *Mycobacterium gordonae* complex is one of the most prevalent populations in aquatic habitats across multiple regions [2,3]. These organisms are frequently regarded as environmental organisms with opportunistic pathogenic potential. However, pulmonary infections have also been reported numerous times [4,6]. *M. gordonae*, the first recognized species of the *M. gordonae* complex, is a slow-growing scotochromogenic bacterium. It has been classified into four clusters based on PCR restriction analysis of the RNA polymerase *rpoB* gene sequence [7]. In recent years, several novel species closely related to the *M. gordonae* complex have been introduced, including *Mycobacterium paragordonae*, *Mycobacterium vicinigordonae* and *Mycobacterium kiyosense* [8,10]. These species share 99% sequence identity in their 16S rRNA genes with *M. gordonae*, making them difficult to distinguish via conventional PCR-based methods.

In a previous study, two *M. gordonae*-like strains associated with human skin infection were isolated in Jiangsu, China [11]. The two strains were previously identified as *M. gordonae* by 16S rRNA gene sequence alignment. However, whole-genome sequence analysis revealed that these strains represented novel species within the *M. gordonae* complex.

## Isolation of the two strains

The strain X7091^T^ was isolated from a nodule that had existed for 5 years on the anterior aspect of a patient's lower leg reported in 2021. Our analysis of whole-genome sequence data revealed that the strain X7091^T^ represented a novel species within the *M. gordonae* complex [11]. Recently, we re-evaluated a previously identified ‘*M. gordonae*’ strain, Z3061^T^, which was originally classified based on 16S rRNA gene sequence alignment in our hospital in 2017. The strain Z3061^T^ was isolated from a female patient presenting with facial ulcers and nosebleeds [12]. Whole-genome sequencing and analysis support its classification as a novel species in the *M. gordonae* complex. Here, we analysed the genomic and phenotypic characteristics of X7091^T^ and Z3061^T^.

## Morphological, physiological and biochemical phenotypes

The strains Z3061^T^ and X7091^T^ were isolated from clinical samples cultured on Löwenstein–Jensen medium (L–J medium) at 32 °C for 19 and 21 days, respectively, until visible colonies appeared. We examined the biochemical phenotypes of the two strains, as well as *M. gordonae* ATCC 14470^T^, by using a panel of assays performed in independent duplicates [13]. Given that the type strains of *M. paragordonae* 49061^T^ (=JCM 18565^T^=KCTC 29126^T^) and *M. vicinigordonae* 24^T^ (=DSM 105979^T^=CMCC 93559^T^) were unavailable, we included data from previous studies [8,10]. Ziehl–Neelsen staining was conducted on the organisms. Colony morphology, pigment production and growth at 25, 37 and 45 °C were assessed on L–J medium and 7H10 agar plates supplemented with OADC. Biochemical tests including nitrate reductase, arylsulphatase assay on days 3 and 14, heat-stable catalase (pH 7, 68 °C), Tween 80 hydrolysis and urease test were conducted. Inhibition tests with 5% sodium chloride and the ability to grow on MacConkey agar without crystal violet were also carried out.

The strains Z3061^T^ and X7091^T^ appeared as short rod-shaped, acid-fast bacilli under microscopy and produced pigment in the dark. Vigorous growth was observed at 25 and 37 °C. Colonies of the strains X7091^T^, Z3061^T^ and *M. gordonae* ATCC 14470^T^ were orange, smooth and moist in appearance. Detailed biochemical characteristics of the type strains within the *M. gordonae* complex are listed in Table 1. All species shared similar biochemical phenotypes. A negative result of the heat-stable catalase test (pH 7, 68 °C) may distinguish X7091^T^ from the other species.

**Table 1.** Biochemical characteristics of the strains *Mycobacterium camsae* X7091^T^ and *Mycobacterium pumcae* Z3061^T^ and other type strains of the *M. gordonae* complex Symbols: +, positive/growth; −, negative/no growth; ±, variable; \, no data available. Data sources: biochemical data for the compared type strains were compiled from previous publications [8,10]. Strains 1 and 2 were obtained in this study. Strains: 1, *M. camsae* X7091^T^; 2, *M. pumcae* Z3061^T^; 3, *M. kiyosense* IWGMT90018-18076^T^ [10]; 4, *M. vicinigordonae* 24^T^ [9]; 5, *M. paragordonae* JCM 18565^T^ [8,9]; 6, *M. gordonae* ATCC 14470^T^ [10].

Characteristic	1	2	3	4	5	6
Growth on L–J media at:						
25 °C	+	+	\	+	\	+
37 °C	+	+	\	+	\	+
45 °C	−	−	\	−	\	−
Growth on 7H10 agar plates at:						
25 °C	+	+	+	+	+	+
37 °C	+	+	+	+	−	+
45 °C	−	−	−	−	−	−
Detectable growth:						
<7 days	±	±		±	−	±
>7 days	+	+	+	+	+	+
Colony colour	Orange	Orange	Orange	Pale yellow	Orange	Yellow
Colony morphology	Smooth	Smooth	Smooth	Smooth	Smooth	Smooth
Arylsulphatase (14 days)	+	+	+	+	+	±
Heat-stable catalase (pH 7, 68 °C)	−	+	+	+	+	+
Tween hydrolysis (<5 days)	−	−	−	−	−	−
Urease	−	−	−	−	+	−
Nitrate reduction	−	−	−	−	−	−
Growth with 5% NaCl	−	−		−	−	−
Growth on MacConkey agar without crystal violet	−	−		−	−	−

Spectra were obtained using a matrix-assisted laser desorption ionization-time of flight MS system (EXS 3600, Zybio, Chongqing, China). The instrument was calibrated before data acquisition. About 1 µL of protein sample was extracted from 5 mg of solid cultures of the strains X7091^T^, Z3061^T^ and *M. gordonae* ATCC 14470^T^ via acid extraction, following the manufacturer’s instruction. Each sample was spotted in parallel across 8 target areas, and 3 valid spectra were recorded per spot, resulting in a total of 24 spectra per sample. Identification scores were obtained by matching against a commercial database (EXS 3600 v2.0.0.0). All strains were identified as *M. gordonae*. According to the manufacturer’s interpretation criteria, scores for *M. gordonae* ATCC 14470^T^, X7091^T^ and Z3061^T^ ranged from 2.05 to 2.30 (Fig. S1, available in the online Supplementary Material) [14].

## Drug resistance test

Drug susceptibility tests were performed using commercial nontuberculous mycobacterium drug sensitivity kits (BASO, Zhuhai, China) based on a standardized microdilution method in Middlebrook 7H9 medium [15]. This study adhered to the prescribed testing procedures, with quality control ensured using *Mycobacterium peregrinum* ATCC700686 and *Staphylococcus aureus* ATCC29213. The strain X7091^T^ was susceptible to linezolid, ciprofloxacin, rifampin, trimethoprim–sulfamethoxazole, moxifloxacin, clarithromycin, amikacin and rifabutin, but it was resistant to meropenem, tobramycin, amoxicillin–clavulanate and imipenem. The strain Z3061^T^ was susceptible to linezolid, cefoxitin, rifampin, trimethoprim–sulfamethoxazole, moxifloxacin, clarithromycin, amikacin and rifabutin, but it was resistant to meropenem and amoxicillin–clavulanate (Table S1).

## Genomic characteristics and phylogenetic analysis

Genomic DNA was extracted from bacterial subcultures stored at −80 °C using the QIAamp DNA Blood Mini Kit (Qiagen, Manchester, UK) after lysis with 20 mg ml^−1^ lysozyme for 30 min. X7091^T^ was sequenced using the PacBio RS II platform and the Illumina HiSeq 4000 platform. The PacBio platform used four SMRT cells equipped with Zero-Mode Waveguide arrays to produce the subreads. PacBio subreads shorter than 1 kb were removed. Sequencing yielded 348,138 subreads with an N50 of 10,329 bp, achieving 37.10-fold coverage. Draft genomic unitigs were assembled using the Celera Assembler v8.3 [16]. The strain Z3061^T^ was sequenced using the Nanopore PromethION platform and the Illumina NovaSeq PE150. Sequencing yielded 115,013 reads with N50 of 11,921 bp, achieving 161.03-fold coverage. Unicycler v0.4.8 was used to assemble data from the PE150 and Nanopore platforms [17].

The quality of the assembled genomes was assessed using CheckM v1.2.3 with the database. Both strains showed nearly complete genomes (Z3061^T^, 100.00%; X7091^T^, 99.86%) with minimal contamination (<1.3%). Prophage prediction was performed using PHAST v1.6, and CRISPR regions were identified using CRISPRFinder v4.2 [18,19]. Genomic features of X7091^T^ and Z3061^T^ are summarized in Table 2. The strain X7091^T^ possessed a complete 6.98 Mb (7,319,570 bp) genome with a G+C content of 66.66 mol%, including a 216,348 bp plasmid (G+C content=64.60mol%). A total of 6,704 coding genes, 48 tRNA genes, 1 5sRNA gene, 1 16sRNA gene, 1 23sRNA gene and 1 small RNA gene were predicted. The strain Z3061^T^ had a 6.48 Mb (6,790,973 bp) genome (G+C content=67.27mol%). A total of 6,018 coding genes, 46 tRNA genes and 3 rRNA genes were predicted.

**Table 2.** Genomic information of X7091^T^ and Z3061^T^

	X7091^T^	Z3061^T^
Genome length (bp)	7,319,570	6,790,973
Genome GC (%)	66.66	67.27
Coding sequences	6,704	6,108
Number of plasmid	1	0
Number of prophage	8	0
Number of CRISPR	13	13
tRNA (% in genome)	48	46
5 s rRNA (% in genome)	1	1
16 s rRNA (% in genome)	1	1
23 s rRNA (% in genome)	1	1
sRNA (% in genome)	1	0

**Fig. 1.**
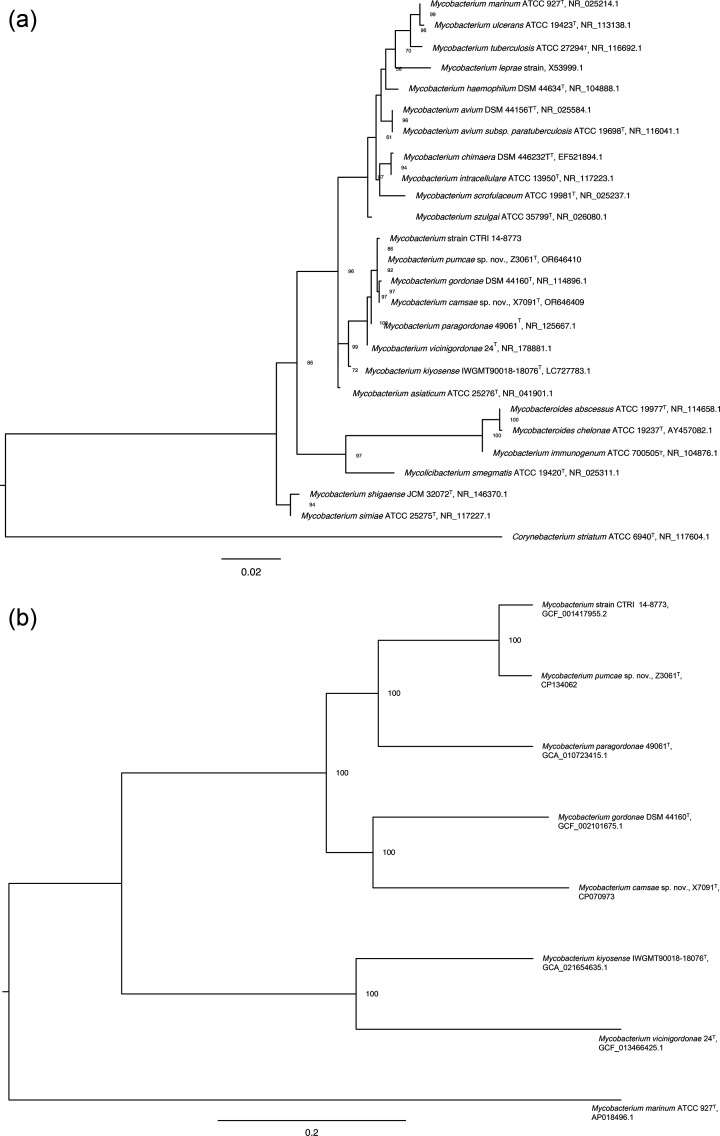
Phylogeny of the type strains within the *M. gordonae* complex and related species based on 16S rRNA gene sequences (**a**) and core gene alignment (**b**).

Although both strains shared >99% 16S rRNA gene identity with *M. gordonae* strains, analysis of housekeeping genes revealed distinct differences, suggesting species-level divergence. Strain Z3061^T^ showed 98.00% (*hsp65*) and 97.58% (*rpoB*) identity with *M. paragordonae* 49061, whereas strain X7091^T^ exhibited 98.67% hsp65 identity with *M. gordonae* DWMJ-1727A1 and 97.17% *rpoB* identity with *M. paragordonae* 49061. A phylogenetic tree utilizing 16S rRNA gene sequences from the strains X7091^T^ and Z3061^T^ was constructed via IQ-TREE v2.1.2, using the maximum likelihood method under the TN+F+R2 model with 1,000 bootstrap replications (Fig. 1a) [20]. Core gene alignments of X7091^T^, Z3061^T^ and other type strains of the *M. gordonae* complex were produced by Roary v3.13.0 and analysed by RAxML v8.2.12 under a general time-reversible plus gamma model with 1,000 bootstrap replications (Fig. 1b) [21,22]. These phylogenetic analyses suggested that X7091^T^ and Z3061^T^ each formed a distinct branch within the *M. gordonae* complex, separated from other type strains.

To clarify the taxonomic relationship of the strains X7091^T^ and Z3061^T^, we conducted comparative analyses against type strain genomes of the *M. gordonae* complex retrieved from the NCBI (https://www.ncbi.nlm.nih.gov/). The average nucleotide identity (ANI) was calculated using FastANI v2.2.23 [23]. *In silico* DNA–DNA hybridization (isDDH) values were estimated using the Type Strain Genome Server with formula *d*_4_ [24,25]. Comprehensive pairwise ANI and isDDH (formula *d*_4_) values comparing the two strains with all relevant type strains are summarized in Table 3. The ANI (<90%) and isDDH (<40%) values between the strains X7091^T^ and Z3061^T^ and other type strains of the *M. gordonae* complex were below the threshold for species delineation (ANI ≥95 % and isDDH ≥70%) [26].

**Table 3.** ANI (upper triangle matrix) and isDDH (lower triangle matrix) values (per cent) among X7091^T^ and Z3061^T^ and other type strains of the *M. gordonae* complex All isDDH values were calculated using the TYGS-recommended formula *d*_4_. na, not applicable. Strains: 1, *M. kiyosense* IWGMT90018-18076^T^; 2, *M. vicinigordonae* 24^T^; 3, *M. paragordonae* JCM 18565^T^; 4, *M. gordonae* DSM 44160^T^; 5, *M. camsae* X7091^T^; 6, *M. pumcae* Z3061^T^.

Species	1	2	3	4	5	6
1	na	86.6	82.1	81.7	81.4	82.1
2	31.6	na	81.0	80.6	80.4	81.0
3	23.9	22.8	na	86.8	86.4	89.5
4	24.1	22.8	31.8	na	87.7	87.0
5	23.8	22.7	31.4	34.6	na	86.7
6	24.0	22.8	37.1	32.2	31.5	na

Taxonomic conclusions were drawn based on the abovementioned phylogenetic and genomic evidence. All these results demonstrated that the strains Z3061^T^ and X7091^T^ were distinct from known species and represented two novel species of the *M. gordonae* complex. We propose *Mycobacterium camsae* as the name for the strain X7091^T^ and *Mycobacterium pumcae* as the name for the strain Z3061^T^. We also found that the strain CTRI 14-8773, previously obtained from the sputum of a patient with suspected pulmonary tuberculosis in Russia (2016), was classified within *M. pumcae* [11,27].

To identify genomic variations between the two strains, homologous gene identification and clustering were performed using the OrthoVenn3 tool [28]. Orthologous clustering analysis of the protein sequences of X7091^T^ and Z3061^T^ yielded a total of 4,529 protein clusters. Among them, 3,696 (81%) were single-copy gene clusters, indicating that the core genomic components of both strains were highly conserved. X7091^T^ and Z3061^T^ exhibited 884 (13.37%) and 539 (8.95%) singletons, respectively. GO enrichment analysis revealed that the specific proteins of X0791 were predominantly enriched in functional categories such as ‘pathogenesis’, ‘cell surface’ and ‘plasma membrane’. The specific proteins of Z3061^T^ were enriched in categories such as ‘protein serine/threonine kinase activity’ and ‘cell wall’ (Fig. S2). Virulence genes were annotated using the VFDB database in Abricate v1.0.1, indicating that the two new species and *M. vicinigordonae* 24^T^ lacked the genes encoding *eccD3*, *eccD5*, *mgtC* and *mycP3*, which differentiated them from other *M. gordonae*-like species [29,30].

## Description of *Mycobacterium camsae* sp. nov.

*Mycobacterium camsae* (cam’sae. N.L. gen. n. *camsae*) was derived from CAMS in the Chinese Academy of Medical Sciences and Peking Union Medical College, referring to the institution where the bacterial isolate was taxonomically characterized and the institutional affiliation of the research team responsible for its discovery.

This bacterium is acid-fast and exhibits a short-rod morphology. The strain can be cultivated at 25–37 °C. Growth is not observed at 45 °C. The colonies are orange, smooth and moist, exhibiting scotochromogenic properties on L–J culture medium and Middlebrook 7H10 agar medium. *M. camsae* does not produce catalase (pH 7, 68 °C), reduce nitrate or hydrolyse Tween 80. It is negative for urease activity. Growth can be observed on MacConkey agar and on 7H10 agar medium for the 14-day arylsulphatase test. Minimal growth occurs in the presence of 5% NaCl. The type strain X7091^T^ (=CGMCC 1.90336^T^=JCM 37414^T^) was isolated from a skin infection biopsy sample collected at the Hospital for Skin Diseases, Institute of Dermatology, Chinese Academy of Medical Sciences and Peking Union Medical College in Jiangsu Province, PR China. The DNA G+C content of the genome is 66.66 mol%. The genome size of the type strain is 6.98 Mb. This species can be distinguished from its closest relatives by ANI ≤87.7% and isDDH ≤34.6% (Table 3). The GenBank accession numbers were GCA_017086405.1 (genome assembly), NZ_CP070973 (chromosome), NZ_CP070974 (plasmid) and OR646409 (nearly full-length 16S rRNA gene sequence).

## Description of *Mycobacterium pumcae* sp. nov.

*Mycobacterium pumcae* (pum'cae, N.L. gen. n. *pumcae*) pertains to PUMC, referring to the Chinese Academy of Medical Sciences and Peking Union Medical College, the institution where the bacterial isolate was taxonomically characterized and the institutional affiliations of the research team responsible for its discovery.

This bacterium exhibits acid-fastness and possesses short-rod morphology. The strain is cultivatable at 25–37 °C. Growth is not observed at 45 °C. Colonies are orange, smooth and moist, exhibiting scotochromogenic properties on L–J culture medium and Middlebrook 7H10 agar medium. Growth is observed on 7H10 agar medium for the 14-day arylsulphatase test. *M. pumcae* produces catalase (pH 7, 68 °C), and it does not reduce nitrate or hydrolyse Tween 80. It is negative for urease activity. Growth is observed on MacConkey agar. Minimal growth occurs in the presence of 5% NaCl. The type strain Z3061^T^ (=CGMCC 1.90337^T^=JCM 37415^T^) was isolated from a skin infection biopsy sample collected at the Hospital for Skin Diseases, Institute of Dermatology, Chinese Academy of Medical Sciences and Peking Union Medical College in Jiangsu Province, PR China. The G+C content of DNA is 67.27 mol%. The genome size of DNA of the type strain is 6.48 Mb. This species can be distinguished from its closest relatives by ANI ≤89.5% and isDDH ≤37.1% (Table 3). The GenBank accession numbers were GCA_031583025.1 (genome assembly for chromosome CP134062) and OR646410 (nearly full-length 16S rRNA gene sequence).

## Supplementary material

10.1099/ijsem.0.006960 Uncited Supplementary Material 1.

## Abbreviations

ANI: average nucleotide identity
isDDH: *in silico* DNA–DNA hybridization
L–J medium: Löwenstein–Jensen medium
