# Supplementary material for: Mycobacterium camsae sp. nov. and Mycobacterium pumcae sp. nov., two species isolated from human skin infection
Source: Int J Syst Evol Microbiol. 2025 Nov 14;75(11):006960. doi: 10.1099/ijsem.0.006960 (PMC12617827; doi:10.1099/ijsem.0.006960)
Supplement: Uncited Supplementary Material 1. [file ijsem-75-06960-s001.pdf]

**Supplementary Table 1**. Antimicrobial susceptibility testing result of X7091$^T$ and Z3061$^T$. NA, Not applicable. Interpretive criteria are based on CLSI guidelines [15].

| Antibiotic | Susceptible (S) (μg/mL) | Intermediate (I) (μg/mL) | Resistant (R) (μg/mL) | MIC of X7091$^T$ (μg/mL) | Interpretation of X7091$^T$ | MIC of Z3061$^T$ (μg/mL) | Interpretation of Z3061$^T$ |
|---|---|---|---|---|---|---|---|
| Meropenem | ≤4 | 8-16 | ≥32 | >64 | R | 8 | I |
| Linezolid | ≤8 | 16 | ≥32 | ≤1 | S | 4 | S |
| Ciprofloxacin | ≤1 | 2 | ≥4 | 1 | S | 1 | I |
| Cefoxitin | ≤16 | 32-64 | ≥128 | 32 | I | 16 | S |
| Tobramycin | ≤2 | 4 | ≥8 | 16 | R | 4 | I |
| Amoxicillin clavulanate potassium | ≤8/4 | 16/8 | ≥32/16 | >64/32 | R | >64/32 | R |
| minocycline | ≤1 | 2-4 | ≥8 | 4 | I | 4 | I |
| Rifampin | ≤1 | — | ≥2 | 1 | S | 1 | S |
| TMP-SMZ | ≤2/38 | — | ≥4/76 | ≤0.25/4.8 | S | ≤ 0.25/4.8 | S |
| Moxifloxacin | ≤1 | 2 | ≥4 | 0.5 | S | ≤0.25 | S |
| Imipenem | ≤4 | 8-16 | ≥32 | >64 | R | 8 | I |
| Tigecycline | ≤4 | — | ≥4 | 2 | NA | 1 | NA |
| Doxycycline | ≤1 | 2-4 | ≥8 | 4 | I | 4 | I |
| Clarithromycin | ≤2(RGM)<br>≤8(SGM) | 4<br>16 | ≥8<br>≥32 | ≤1 | S | ≤1 | S |
| Amikacin | ≤16 | 32 | ≥64 | ≤2 | S | ≤2 | S |
| Rifabutin | ≤2 | — | ≥4 | ≤0.5 | S | ≤0.5 | S |

**Supplementary Figure 1.** MALDI–TOF MS profiles of X7091$^{T}$(A), Z3061$^{T}$(B), and *Mycobacterium gordonae* ATCC 14470$^{T}$(C). The x-axis represents the mass-to-charge ratio (m/z) measured in Daltons (Da), the y-axis represents the relative intensity (in %) of the signal..

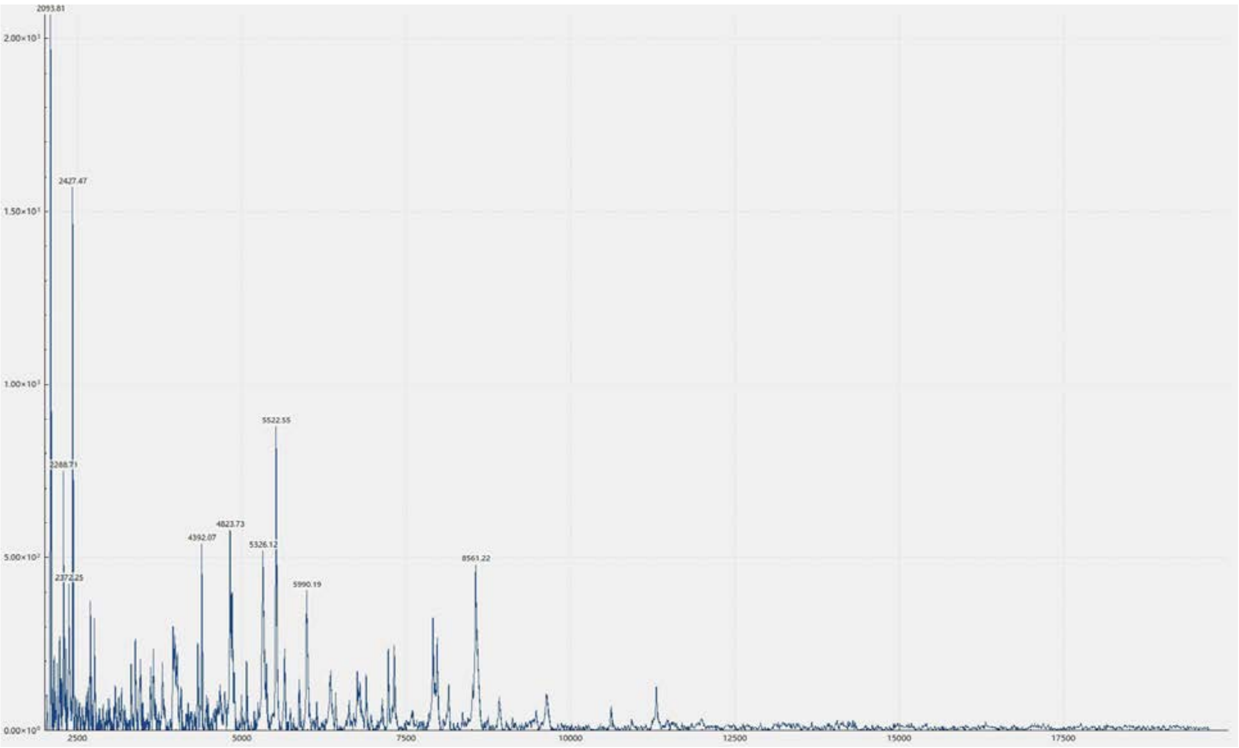


(A)

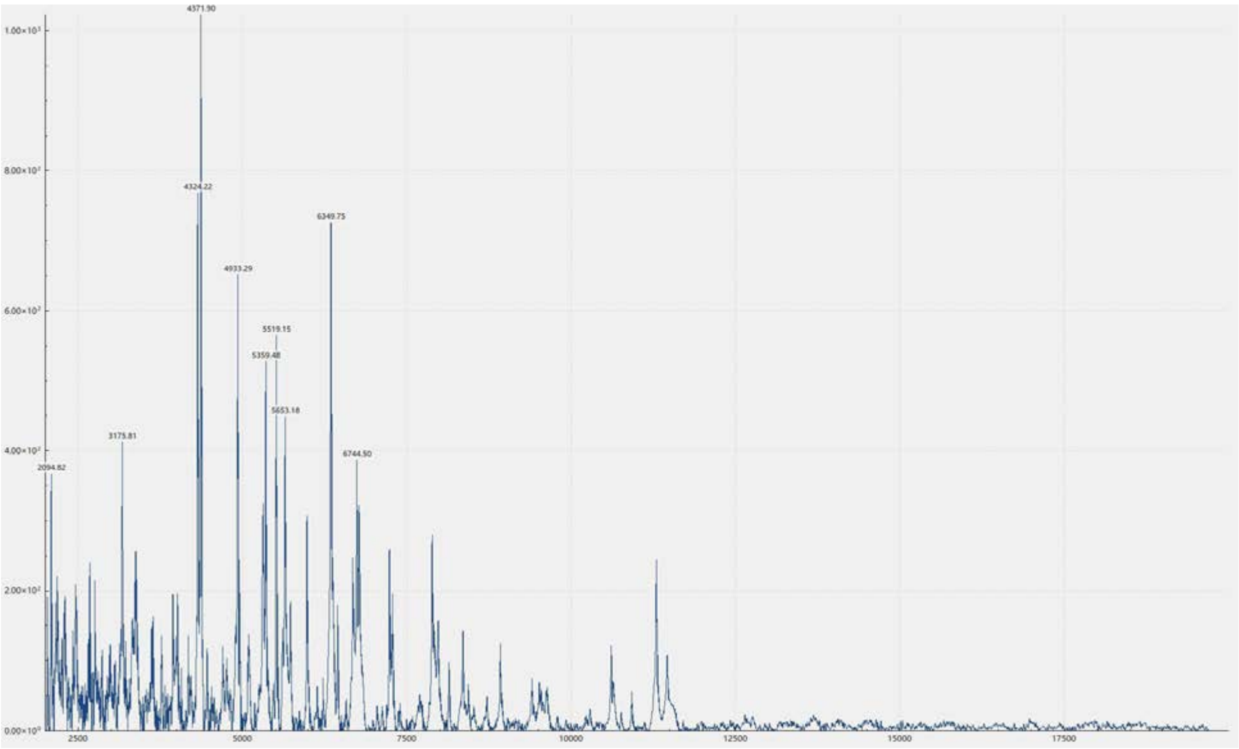


(B)

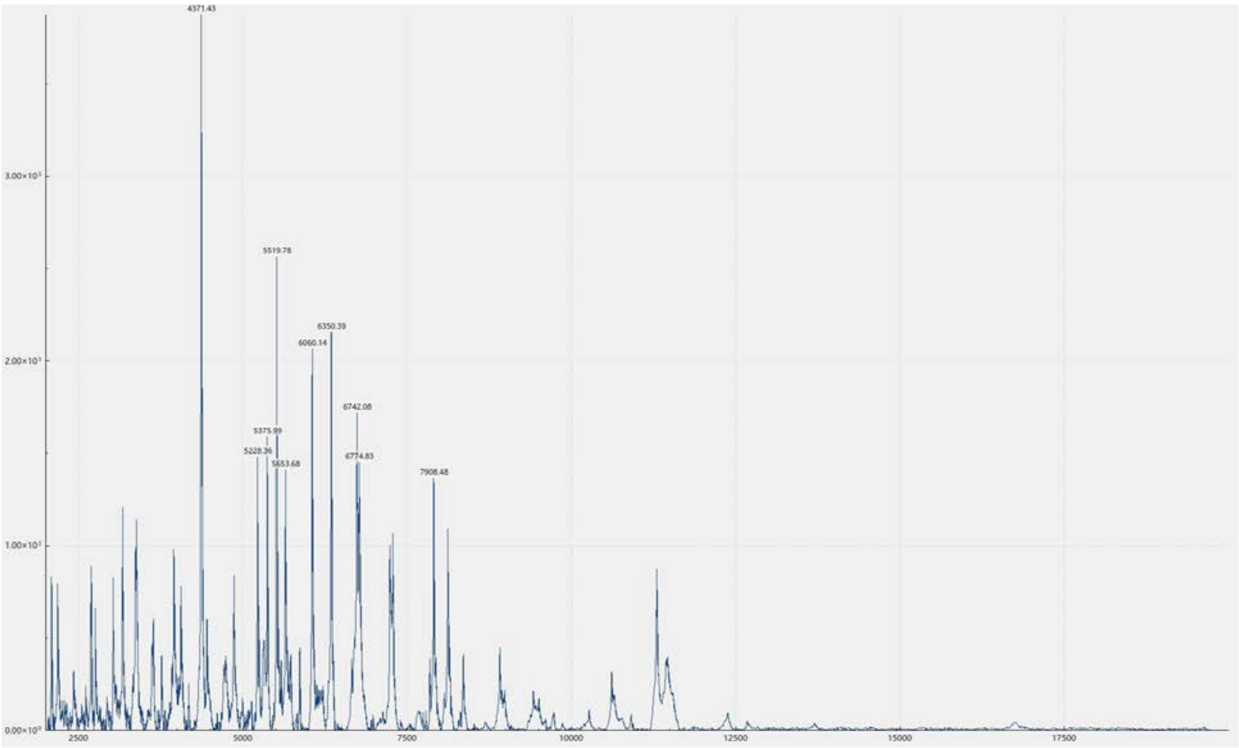
4371.43
5519.78
6350.39
6060.14
6742.08
5375.99
5228.36
5653.68
6774.83
7908.48
3.00×10³
2.00×10³
1.00×10³
0.00×10⁰
2500
5000
7500
10000
12500
15000
17500

(C)

**Supplementary Figure 2.** Bubble chart of GO enrichment result of the unique protein clusters in X7091[T] and strain Z3061[T].

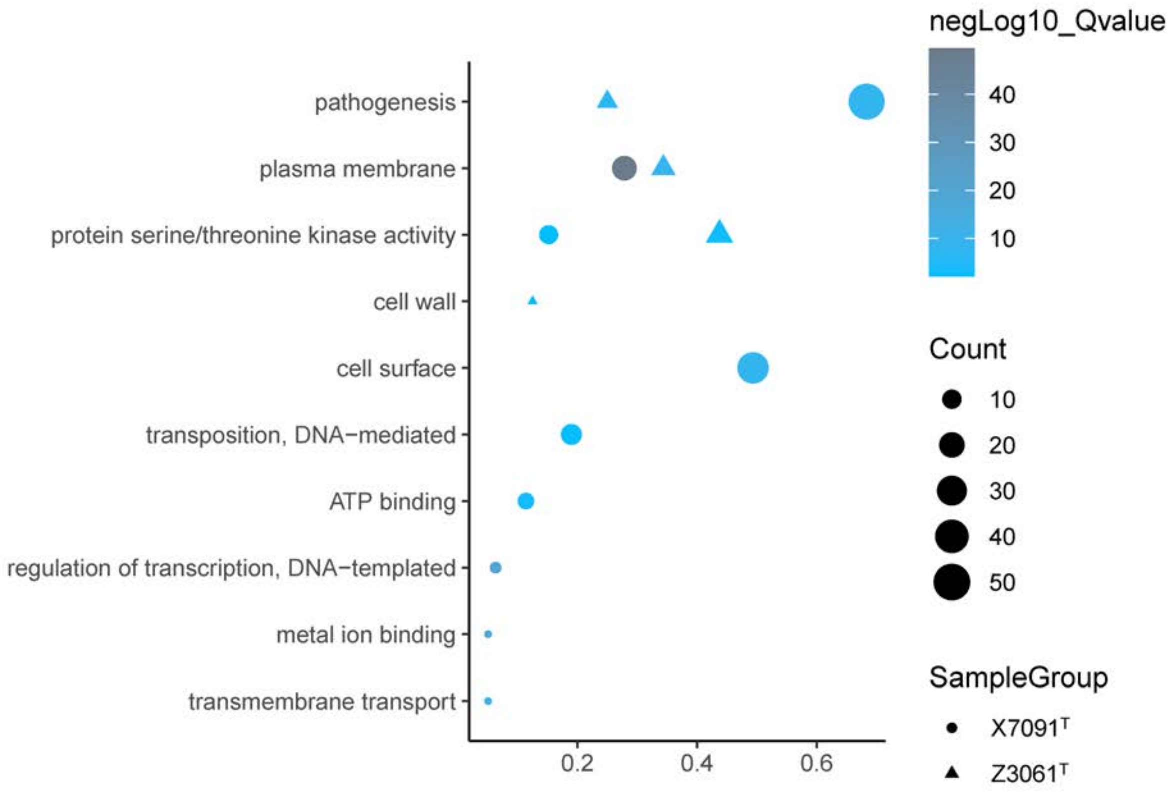

**Supplementary Table 2**. Pairwise in-silico DNA–DNA hybridization (isDDH) values between the genomes of strains X7091[T] (CP134062), Z3061[T] (NZ_CP070973) and the type strains of most closely related species calculated using the Type Strain Genome Server (TYGS) [24].

Three different formulas used Genome BLAST Distance Phylogeny (GBDP) in TYGS for analyses are: $d_0$, sum of all high-scoring segment pairs (HSPs) divided by total genome length; $d_4$, sum of all identities found in HSPs divided by overall HSP length, independent of genome length; $d_6$, number of all identities within HSPs divided by total genome length; C.I., 95% confidence intervals [25].

| Query strain | Subject strain | dDDH ($d_0$, in %) | C.I. ($d_0$, in %) | dDDH ($d_4$, in %) | C.I. ($d_4$, in %) | dDDH ($d_6$, in %) | C.I. ($d_6$, in %) |
|---|---|---|---|---|---|---|---|
| CP134062 | *Mycobacterium paragordonae* JCM 18565 | 69.5 | [65.5 - 73.1] | 37.1 | [34.6 - 39.6] | 61.8 | [58.5 - 65.0] |
| CP134062 | *Mycobacterium gordonae* DSM 44160 | 57.3 | [53.7 - 60.8] | 32.2 | [29.8 - 34.7] | 50.5 | [47.4 - 53.5] |
| CP134062 | *Mycobacterium kiyosense* IWGMT90018-18076 | 31 | [27.6 - 34.6] | 24 | [21.7 - 26.5] | 28.3 | [25.4 - 31.4] |
| CP134062 | *Mycobacterium asiaticum* DSM 44297 | 34.1 | [30.7 - 37.6] | 23.5 | [21.2 - 26.0] | 30.4 | [27.4 - 33.5] |
| CP134062 | *Mycobacterium vicinigordonae* 24[T] | 27.8 | [24.4 - 31.4] | 22.8 | [20.5 - 25.3] | 25.6 | [22.8 - 28.7] |
| NZ_CP070973 | *Mycobacterium gordonae* DSM 44160 | 53.2 | [49.8 - 56.7] | 34.6 | [32.2 - 37.1] | 48.5 | [45.4 - 51.5] |
| NZ_CP070973 | *Mycobacterium paragordonae* JCM 18565 | 52 | [48.5 - 55.5] | 31.4 | [29.0 - 33.9] | 46.3 | [43.3 - 49.3] |
| NZ_CP070973 | *Mycobacterium kiyosense* IWGMT90018-18076 | 27.7 | [24.4 - 31.3] | 23.8 | [21.5 - 26.3] | 25.8 | [22.9 - 28.9] |
| NZ_CP070973 | *Mycobacterium asiaticum* DSM 44297 | 29.2 | [25.8 - 32.8] | 23.3 | [21.1 - 25.8] | 26.8 | [23.9 - 29.9] |
| NZ_CP070973 | *Mycobacterium vicinigordonae* 24[T] | 24.9 | [21.6 - 28.6] | 22.7 | [20.4 - 25.2] | 23.4 | [20.6 - 26.5] |
